# Ventilator-associated pneumonia in patients undergoing major heart surgery: an incidence study in Europe

**DOI:** 10.1186/cc7896

**Published:** 2009-05-22

**Authors:** Javier Hortal, Patricia Muñoz, Gregorio Cuerpo, Hector Litvan, Peter M Rosseel, Emilio Bouza

**Affiliations:** 1 Anaesthesia Department. Hospital General Universitario Gregorio Marañón. Dr. Esquerdo 46 – 28007 Madrid, Spain; 2 Clinical Microbiology and Infectious Diseases Department. Hospital General Universitario Gregorio Marañón. Dr. Esquerdo 46 – 28007 Madrid, Spain; 3 Cardiac Surgery Department. Hospital General Universitario Gregorio Marañón. Dr. Esquerdo 46 – 28007 Madrid, Spain; 4 Anaesthesia Department. Hospital Sant Creu i Sant Pau. Sant Antoni Maria Claret, 167 – 08025 Barcelona, Spain; 5 Anaesthesia and Critical Care Department. Thoraxcenter Amphia. Galderseweg 81 – 4836AE Breda, Holland; 6 Centro de Investigación Biomédica en Red de Enfermedades Respiratorias, Fundación Caubet-Cimera, Recinto Hospital Joan March, Carretera Soller Km 12, 07110, Bunyola, Mallorca, Spain

## Abstract

**Introduction:**

Patients undergoing major heart surgery (MHS) represent a special subpopulation at risk for nosocomial infections. Postoperative infection is the main non-cardiac complication after MHS and has been clearly related to increased morbidity, use of hospital resources and mortality. Our aim was to determine the incidence, aetiology, risk factors and outcome of ventilator-associated pneumonia (VAP) in patients who have undergone MHS in Europe.

**Methods:**

Our study was a prospective study of patients undergoing MHS in Europe who developed suspicion of VAP. During a one-month period, participating units submitted a protocol of all patients admitted to their units who had undergone MHS.

**Results:**

Overall, 25 hospitals in eight different European countries participated in the study. The number of patients intervened for MHS was 986. Fifteen patients were excluded because of protocol violations. One or more nosocomial infections were detected in 43 (4.4%) patients. VAP was the most frequent nosocomial infection (2.1%; 13.9 episodes per 1000 days of mechanical ventilation). The microorganisms responsible for VAP in this study were: *Enterobacteriaceae *(45%), *Pseudomonas aeruginosa *(20%), methicillin-resistant *Staphylococcus aureus *(10%) and a range of other microorganisms. We identified the following significant independent risk factors for VAP: ascending aorta surgery (odds ratio (OR) = 6.22; 95% confidence interval (CI) = 1.69 to 22.89), number of blood units transfused (OR = 1.08 per unit transfused; 95% CI = 1.04 to 1.13) and need for re-intervention (OR = 6.65; 95% CI = 2.10 to 21.01). The median length of stay in the intensive care unit was significantly longer (*P *< 0.001) in patients with VAP than in patients without VAP (23 days versus 2 days). Death was significantly more frequent (*P *< 0.001) in patients with VAP (35% versus 2.3%).

**Conclusions:**

Patients undergoing aortic surgery and those with complicated post-intervention courses, requiring multiple transfusions or re-intervention, constitute a high-risk group probably requiring more active preventive measures.

## Introduction

Patients undergoing major heart surgery (MHS) represent a special subpopulation at risk for nosocomial infections. Postoperative infection is the main non-cardiac complication after MHS and has been clearly related to increased morbidity, use of hospital resources and mortality [1,2].

Ventilator-associated pneumonia (VAP) is the most common infection in patients admitted to intensive care units (ICUs) [3,4] and is a leading cause of morbidity and mortality [5,6]. The situation of VAP in patients undergoing MHS has been assessed only from the perspective of single institutions with the bias of the case-mix at a particular centre [1,7-10]. Our group led the collection of retrospective data of VAP in MHS from several European institutions [11], but prospective data from a large group of European centres were lacking.

Our study aims were to determine the incidence, aetiology, risk factors and outcome of VAP in a large sample of patients who have undergone MHS in Europe.

## Materials and methods

Our study is a joint venture between the European Study Group of Nosocomial Infection (ESGNI), the European Society of Clinical Microbiology and Infectious Diseases (ESCMID) and the European Working Party of Cardiothoracic Intensivists (EWCI). The Ethics Committee of Hospital General Universitario Gregorio Marañón (Madrid, Spain) approved the study and indicated that individual informed consent was not necessary in this study because no intervention was performed and confidentiality was respected.

Our study (ESGNI 09 study) was a prospective (one-month enrolment) analysis of patients undergoing MHS in Europe who developed suspicion of VAP. During a one-month period participating units submitted a protocol of all patients admitted to their units who had undergone MHS. Specific variables on VAP diagnosis and evolution were included.

Units and investigators willing to participate sent data regarding the type of hospital, (public or private, teaching or non-teaching, total population surveyed, number of beds, and the number of hospital admissions for 24 hours or longer during the month of the study) and data regarding the ICU used for postoperative care of MHS patients in each institution (ICU specific for MHS or mixed with other types of patients and number of beds available).

Individual data for patients admitted to European postsurgical ICUs included: hospital admission date, sex, age, prior illnesses, clinical characteristics of the patient and New York Heart Association (NYHA) functional class. Patients' underlying diseases were classified according to the criteria of McCabe and Jackson [12] as rapidly fatal, ultimately fatal and non-fatal; their morbidity scores were based on the Charlson co-morbidity index [13]. The American Society of Anesthesiologists physical status grading system [14] and EuroSCORE [15] were used to value surgical risk.

Data regarding the surgical procedure included type of indication (elective, urgent or emergent), type of surgical procedure, duration (from the skin incision until closure), time on cardiopulmonary bypass, aortic cross-clamp time, surgical antimicrobial prophylaxis, transfusion needs, overall period with chest drainages, number of reinterventions and need for inotropic support, intra-aortic balloon or circulatory assistance. Surgical prophylaxis was performed according to each centre's protocol.

Recorded postsurgical events included ICU admission and discharge date, days spent on mechanical ventilation, preventive methods for VAP, type of nosocomial infection and patient evolution. If the patient had VAP, a specific part of the questionnaire was completed including criteria for diagnosis, Clinical Pulmonary Infection Score (CPIS) [16], microbiological data (microorganisms causing pneumonia) and outcome.

### Definitions

VAP was diagnosed upon the presence of new and/or progressive pulmonary infiltrates on chest radiograph plus two or more of the following criteria: fever (≥ 38.5°C) or hypothermia (< 36°C), leucocytosis (≥ 12 × 10^9^/L), purulent tracheobronchial secretions or a reduction of partial pressure of arterial oxygen (PaO_2_)/fraction of inspired oxygen (FiO_2_) of 15% or higher in the past 48 hours according to the definitions of the Centers for Disease Control and Prevention [17]. Also, as pneumonia cases we included those patients with a CPIS higher than six [16].

Tracheobronchitis was defined as the presence of purulent tracheobronchial secretions plus two or more of the following criteria: fever (≥ 38.5°C) or hypothermia (< 36°C), leucocytosis (≥ 12 × 10^9^/L), or significant bacteriological counts in respiratory secretions in patients without pulmonary infiltrates suggesting pneumonia on chest radiograph [17]. Cases with either VAP or tracheobronchitis had to be microbiologically confirmed.

The ICUs were classified as specific if more than 95% of their beds were addressed to patients undergoing MHS or as mixed if this criterion was not met.

### Data analysis

Reports from individual centres were sent to the coordinating centre either by regular mail or via the internet. Individual reports were reviewed by one of the authors before being entered into the database and analysed using SPSS Version 12 (SPSS Inc., Chicago, IL, USA).

We expressed continuous variables as the median and interquartile range (IQR) if their distribution was skewed, and discrete variables as percentages. Measures of significance were assessed by univariate and stratified analysis. Continuous variables were analysed by the Mann-Whitney U test, and categorical variables were analysed with Fisher's exact test or the chi-squared test. All statistical tests were two-tailed. The independent contribution of predictor variables for the development of VAP and mortality after MHS was assessed by stepwise logistic regression analysis, and associations between variables expressed as odds ratios (OR) and respective 95% confidence intervals (CI). As candidate variables we included in the model all those which showed univariate significance less than *P *< 0.1. The aim of the study was to find preoperative, operative and immediate postoperative risk factors of VAP. For this reason risk factors were analysed in two models, with and without the inclusion of the number of days of mechanical ventilation. The models were validated by means of the jack-knifing technique [18]. Variables which did not yield the same results in at least 90% of the 20 jack-knifing runs were discarded. No significant first-order interactions were found in the models.

## Results

### Participating institution characteristics

Overall, 25 hospitals in eight different European countries participated in the study (Table 1). The participating institutions were either teaching (88%) or non-teaching hospitals (12%) and the majority were public centres (92%). The distribution of hospitals according to the number of beds was as follows: less than 500 beds (16%), from 500 to 1000 beds (48%) and more than 1000 beds (36%). Overall, these institutions had performed 13,357 (IQR = 303 to 675) MHS procedures during the previous year. Considering that they were responsible for the health care of 18,173,745 people (IQR = 400,000 to 1,200,000) and they had had 996,780 admissions (IQR = 24,900 to 62,500) during the previous year, we can estimate that there were 73.8 MHS interventions per 100,000 population and 13.4 procedures per 1000 hospital admissions in the areas covered by the participant institutions.

**Table 1:** Participating hospitals and countries

**Country**	**Hospitals**	**Patients per hospital**	**Patients per country**
Austria	AKH University Hospital	19	19
Denmark	Rigshospitalet	72	72
France	Albert Michallon	35	35
Italy	Azienda Ospedaliera-Universita di Padova	64	64
The Netherlands	Amphia Hospital	134	134
Spain	Sant Creu i Sant Pau	59	487
	German Trias i Pujol	19	
	Virgen de las Nieves	26	
	Clínico de San Carlos	16	
	Clínico Universitario de Valencia	22	
	Hospital Universitario 12 de Octubre	32	
	Hospital de Cruces	23	
	Mixoeiro	55	
	Puerta de Hierro	24	
	Hospital de la Princesa	23	
	Virgen de la Macarena	39	
	Gregorio Marañón	39	
	Clínica Ruber	13	
	Hospital Universitario de Canarias	20	
	Ruber Internacional	4	
	Hospital la Fe	38	
	Virgen de la Arrixaca	35	
Sweden	Sahlgrens University Hospital	69	69
Switzerland	University Hospital Zurich	67	91
	Centre Hospitalier Universitaire de Vaudois	24	
Totals = 8	25		971

Only 44% of the ICUs surveyed were used specifically for MHS patients and the median number of available beds in these units was 12 (IQR = 10 to 22).

### Population at risk

The number of patients intervened for MHS during the study period in the different participating centres was 986 (Median = 33, IQR = 21 to 58). Fifteen patients were excluded because of protocol violations. Overall, 971 patients remained in the study. General data regarding the population intervened including the demographic and descriptive data of the patients are listed in Table 2. The mean (standard deviation (SD)) age of the patients was 64.22 (12.11) years and the median length of in hospital preoperative stay was two days (IQR = one to seven).

**Table 2:** Preoperative and surgical characteristics of patients who underwent major heart surgery

Characteristic	Global
Preoperative	
Number of patients	971
Mean age in years (SD)	64.1 (12.2)
Sex, male/female	690/281
Underlying conditions (%)	
Myocardial infarction	351 (36.1)
Congestive heart failure	125 (12.9)
Central nervous system disorder	82 (8.4)
Chronic obstructive pulmonary disease	84 (8.7)
Peripheral vascular disease	179 (18.4)
Ulcer disease	51 (5.3)
Diabetes mellitus	114 (11.7)
Renal disease	33 (3.4)
Malignant neoplasm	14 (1.4)
Liver disease	56 (5.8)
Severe pulmonary hypertension	29 (3.0)
Severe ventricular dysfunction	76 (7.9)
Previous cardiac surgery (%)	96 (9.9)
Mean Charlson comorbidity index (SD)	1.6 (1.6)
McCabe and Jackson groups (%)	
1	68 (7.0)
2	689 (71.0)
3	214 (22.0)
New York Heart Association functional class (%)	
I	148 (15.2)
II	290 (29.9)
III	390 (40.2)
IV	143 (14.7)
American Society of Anesthesiologists score (%)	
1	0
2	10 (1.0)
3	673 (69.4)
4	279 (28.7
5	19 (1.9)
EuroSCORE (%)	
Low risk (0 to 2)	213 (21.9)
Moderate risk (3 to 6)	407 (41.9)
High risk (> 6)	351 (36.1)
Surgical	
Indication (%)	
Elective	781 (80.4)
Urgent	148 (15.2)
Emergent	42 (4.3)
Type of surgery (%)	
Valvular replacement	267 (27.5)
CABG	528 (54.4)
Mixed (valvular and CABG)	76 (7.8)
Heart transplantation	14 (1.4)
Aortic surgery	46 (4.7)
Other	40 (4.1)
Mean duration of surgery (minutes) (SD)	233 (96.0)
Mean cardiopulmonary bypass time (minutes) (SD)	110.1 (54.1)
Mean aortic cross-clamp time (minutes) (SD)	71.9 (42.2)

CABG = Coronary artery bypass grafting; SD = standard deviation.

The interventions were classified as elective in 80.4% of the patients, urgent in 15.2% and emergent in 4.3%. The antimicrobial prophylaxis used was cefazolin (37.8%), vancomycin (4.6%), other drugs (57.3%) and none (0.3%). The mean duration of surgery was 233 (96) minutes. Of the 523 patients undergoing coronary artery bypass grafting (CABG), 122 (23.3%) were performed without cardiopulmonary bypass (CPB). The mean CPB time was 110.1 (54.1) minutes and the mean aortic cross-clamp time was 71.9 (42.2) minutes.

Overall, 477 patients (49.1%) were transfused and the median number of units was three (IQR = two to six). The patients needed inotropic support (59.1%), intra-aortic balloon (6.1%) or circulatory assistance (0.5%) because of different degrees of ventricular dysfunction. The median length of stay in the ICU was two days (IQR = one to three).

### Ventilator-associated pneumonia

**Figure 1:**
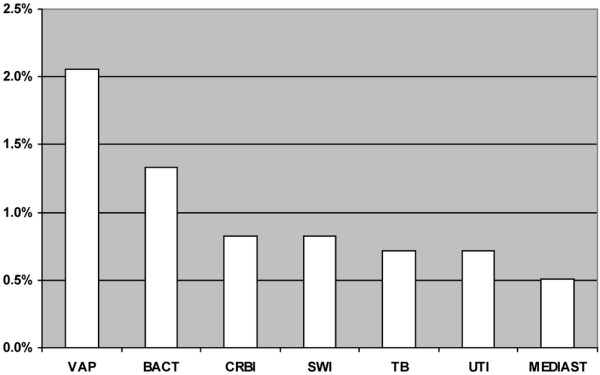
Incidence of nosocomial infections among 971 patients undergoing major heart surgery in Europe. BACT = bacteraemia; CRBI = catheter-related bloodstream infection; MEDIAST = postsurgical mediastinitis; SWI = surgical wound infection; TB = tracheobronchitis; UTI = urinary tract infection; VAP = ventilator-associated pneumonia.

Of the 971 patients undergoing MHS, 43 (4.4%) patients had one or more nosocomial infection (Figure 1). VAP was the most frequent nosocomial infection, with an incidence during the study period of 2.1% (20 of 971 patients). Of these, five patients (25%) had two VAP episodes. The incidence density of VAP in this study was 13.9 episodes per 1000 days of mechanical ventilation.

Overall, only 112 patients (11.5%) required more than 48 hours of mechanical ventilation and 66 (6.8%) more than 72 hours. If we consider only the patients with more than 48 hours of mechanical ventilation, 17.9% (20 of 112) developed VAP and the incidence reached 28.8% among those ventilated for more than 72 hours (19 of 66). The mean CPIS of these patients was 7.5 (1.6) points and the median number of days on mechanical ventilation at the time of VAP was 5.5 days (IQR = 3.0 to 7.7). VAP patients required a median number of 15 days of mechanical ventilation (IQR = 6.2 to 29.7).

In our study, seven patients (0.7%) fulfilled criteria for tracheobronchitis at any time during their clinical course. The incidence rate of tracheobronchitis was 3.7 per 1000 days of mechanical ventilation. If we consider only the patients with more than 72 hours of mechanical ventilation, 10.6% (7 of 66) developed tracheobronchitis. Of these seven patients, two developed a VAP later on. The mean CPIS of the patients with tracheobronchitis was 5.0 (1.7) points and the median number of days on mechanical ventilation at the time of tracheobronchitis was five days (IQR = three to six). Patients with tracheobronchitis required mechanical ventilation during a median of 11 days (IQR = 8.0 to 25.0).

The microorganisms responsible for VAP in this study were: *Enterobacteriaceae *(45%), *Pseudomonas aeruginosa *(20%), methicillin-resistant *Staphylococcus aureus *(10%), *Haemophilus influenzae *(10%), *Serratia *species (10%) and a range of other microorganisms. VAP was polymicrobial in 25% of the episodes (5 among the 20 first cases).

Samples were obtained by means of plain endotracheal aspirate (12; 60%), non-bronchoscopically-guided plugged telescopic catheter (4; 20%), bronchoscopically-guided plugged telescopic catheter (3; 15%) and bronchoscopically-guided bronchoalveolar lavage (1; 5%).

### Risk factors

We analysed preoperative, operative and immediate postoperative risk factors for the development of VAP. In the univariate analysis preoperative factors associated with VAP were (Table 3): mixed ICU (relative risk [RR] = 2.8), peripheral vascular disease (RR = 3), renal disease (RR = 7.9) and American Society of Anesthesiologists score more than 3 (RR = 3.5). For surgical risk factors, the following were associated with VAP: need for inotropic support (RR = 15), need for intra-aortic balloon (RR = 5.5), ascending aorta surgery (RR = 9.7) and median duration of surgery. For postoperative risk factors, the following were associated with VAP: mean number of blood units transfused, need for re-intervention (RR = 12.3) and days of mechanical ventilation until onset of VAP.

**Table 3:** Risk factors for VAP in patients undergoing major heart surgery in Europe in a univariate analysis

**Characteristic**	**VAP (%)** **(n = 20)**	**No VAP (%)** **(n = 951)**	**Relative risk** **(95% confidence interval**	***P *value**
Mixed ICU	12 (60)	330 (34.7)	2.8 (1.1 to 6.9)	0.02
Peripheral vascular disease	8 (40)	171 (18)	3.0 (1.2 to 7.5)	0.01
Renal disease	4 (20)	29 (3)	7.9 (2.5 to 25.2)	0.004
American Society of Anesthesiologists > 3	12 (60)	286 (30)	3.5 (1.4 to 8.6)	0.006
Need for inotropic support	20 (100)	554 (58.3)		< 0.001
Need for intra-aortic balloon	5 (25)	54 (5.7)	5.5 (1.9 to 15.8)	0.005
Ascending aortic surgery	6 (30)	40 (4.2)	9.7 (3.5 to 26.7)	0.001
Median surgery duration in minutes (IQR)	287 (262 to 403)	210 (170 to 260)		< 0.001
Mean number of blood units transfused (SD)	16.8 ± 19.5	2.1 ± 4.4		< 0.001
Need of re-intervention	9 (45)	59 (6.2)	12.3 (4.9 to 31)	< 0.001
Median number of days on mechanical ventilation (IQR)	9.5 (5 to 29)	1 (1 to 1)		< 0.001

ICU = intensive care unit; IQR = interquartile range; SD = standard deviation; VAP = ventilator-associated pneumonia.

Regarding multivariate analysis, two different models were performed, not including or including the days on mechanical ventilation in the model. As for the logistic regression model and considering the number of patients with VAP, only the four variables which yielded stable results in all the runs of the jack-knifing technique were included. With the use of multivariate analysis (first model), we identified the following significant independent risk factors for VAP (Table 4): ascending aorta surgery (OR = 6.22; 95% CI = 1.69 to 22.89), number of blood units transfused (OR = 1.08 per unit transfused; 95% CI = 1.04 to 1.13) and need for re-intervention (OR = 6.65; 95% CI = 2.10 to 21.01). When 'number of days of mechanical ventilation' was included as a covariate in a separate model, significant independent risk factors for VAP were: need for re-intervention (OR = 11.97; 95% CI = 2.76 to 51.81) and days of mechanical ventilation (OR = 1.41 per day of mechanical ventilation; 95% CI = 1.24 to 1.61).

**Table 4:** Risk factors for VAP in patients undergoing major heart surgery in Europe and a multivariate analysis

**Characteristic**	**Odds ratio**	**95% confidence interval**	***P *value**
Ascending aortic surgery	6.22	1.69 to 22.89	0.006
Number of blood units transfused (per unit transfused)	1.08	1.04 to 1.13	< 0.001
Need for re-intervention	6.65	2.10 to 21.01	0.001

### Treatment

Data on antimicrobial management was available from 19 of 20 VAPs. Time elapsed from clinical diagnosis to the start of therapy was classified as: less than 8 hours (9 of 19; 47.4%), 8 to 24 hours (9 of 19; 47.4%) and more than 48 hours (1 of 19; 5.2%). Empirical therapy consisted of one drug (12; 63.2%), two drugs (5; 26.3%) and more than two drugs (2; 10.5%). Empirical therapy was changed in 10 patients (52.6%) due to microbiological data (five patients), absence of clinical response (three patients) or both (two patients). Empirical therapy was considered adequate in 13 patients (68.4%). Definite therapy consisted of one drug (6; 31.6%), two drugs (8; 42.1%) and more than two drugs (5; 26.3%).

### Outcome

The median length of stay in the ICU was significantly longer (*P *< 0.001) in patients with VAP than in patients without VAP (23 vs 2 days). Overall ICU mortality in patients who underwent MHS was 3% (29 of 971). Death was significantly more frequent (*P *< 0.001) in patients with VAP (35% vs 2.3%).

With the use of multivariate analysis, we identified the following significant independent risk factors for mortality: peripheral vascular disease (OR = 3.35, CI = 1.46 to 7.67), intra-aortic balloon (OR = 8.21, CI = 3.38 to 19.94) and need for re-intervention (OR = 3.46, CI = 1.29 to 9.26). VAP was, as well, an independent risk factor for mortality (OR = 8.62, CI = 2.63 to 28.26).

## Discussion

Our multicentre European study confirms that VAP was the main cause of postoperative infection in patients undergoing MHS in several European centres. Our results showed incidence data between that reported from institutions with very different case mixed.

Figures of incidence of nosocomial infections in general ICUs vary from 9 to 37%, mostly depending on the type and severity of illness of that population and the definitions used [19,20]. In patients undergoing MHS, figures of postoperative nosocomial infections range from 9 to 45%, depending also on the type of heart surgery performed [1,21].

VAP is the most common ICU-acquired infection both in general and surgical ICUs [4,6], and that also holds true in patients undergoing MHS [1,7,11,22]; however, rates are very variable and range from 3 to 21.6% [1,7,8,10,21,23-25], probably depending on the different case mix of the individual reporting institutions.

According to the National Nosocomial Infections Surveillance report of 2004, the median rate of VAP in 47 cardiothoracic surgery ICUs was 6.3 (IQR = 2.9 to 12.6) per 1000 ventilation days [26]; however, the proportion of patients undergoing MHS in that population is not clear. In a previous study, several European MHS units retrospectively estimated their incidence of VAP which occurred in 3.8% of all patients undergoing MHS [11]. The present study provides a prospectively collected incidence rate of 2.1% (incidence density of 13.9 of 1000 ventilation days) which is lower than previous results found in similar patients [1,7,8], although it is comparable with those reported in other studies [9,10].

Microorganisms causing VAP vary considerably according to the characteristics of the patients in the different ICU types, the length of hospital stay and intubation. Common pathogens include *P. aeruginosa*, *S. aureus *and *Enterobacteriaceae *[27]. There is no evidence that the microorganisms causing VAP after MHS are substantially different [1,7,9] to those in other types of patients in ICUs. In a paper from Kollef and colleagues [1], 59 of 605 MHS patients developed VAP. *Enterobacteriaceae *(15 cases) and *P. aeruginosa *(9 cases) predominated, as happened in our series. Our series also showed the potential presence of *S. aureus *and particularly the risk of methicillin-resistant isolates. The proportion of polymicrobial VAP ranged from 13 to 55% in different studies [28-30]. In our series 25% of the VAP episodes had more than one microorganism present.

Various risk factors have been associated with the development of VAP in patients undergoing MHS, including the duration of mechanical ventilation, need for reintubation, transfusion needs, empirical administration of broadspectrum antibiotics, type of surgery, age over 60 years, supine position during the first 24 hours, history of chronic obstructive pulmonary disease, NYHA score of 3 or higher and need for mechanical intravascular support [1,7,8,21,23,24,31]. Some of these factors were confirmed in our study, in particular, transfusion needs and type of surgery. At the same time, our study underscores other risk factors such as the need for re-intervention with haemorrhage or cardiac tamponade in the immediate postoperative period. Our study was oriented to find preoperative, intraoperative and immediate postoperative factors amenable to intervention in the population undergoing MHS. Due to this, we decided not to include the variable 'days of mechanical ventilation' in the model because it completely overshadowed the importance of the other variables we specifically wanted to address.

Because of this, we decided to include the variable 'days of mechanical ventilation' in a separate model. After analysing this new model, transfusion needs lost statistical significance.

Most unfortunately, the majority of the variables that significantly predict VAP are not amenable to intervention. In our opinion the use of anticipative or pre-emptive antimicrobial therapy should be explored as one of the few potential interventions to avoid VAP in the high-risk population. It is known that inadequate empirical therapy is associated with an increase in VAP-related mortality, even if it is corrected in the following hours. Singh and colleagues demonstrated that the administration of three days of ciprofloxacin to patients with suspicion of VAP had a very favourable impact on the cost and length of antimicrobial use, and reduced the rate of superinfections and the emergence of resistance [32]. Also, the use of oral decontamination, along with three days of cefotaxime or ceftriaxone, has been demonstrated to have the potential benefit of antimicrobial pre-emptive therapy in patients at high risk of VAP [33,34]. Other potential preventive measures include continuous aspiration of subglottic secretions [35] or the use of polyurethane cuffed tubes [36].

The overall mortality rate for VAP in patients undergoing MHS may be as high as 16 to 57% [1,7,9], but many critically ill patients with VAP die because of their underlying disease rather than of pneumonia. Crude mortality rate of patients with VAP was found to be 35% in our study and attending physicians attributed 13.8% of excess deaths to VAP. However, it should be stated that because multiple comorbidities in these patients, the attribution of mortality to VAP should always be interpreted with caution.

Some limitations of this investigation should be mentioned. Countries and institutions were not randomly selected among the whole continent and the relative weight of the European countries is not equilibrated. However, this study includes 25 centres from eight European countries and constitutes, to our knowledge, the best data available to date to estimate the dimension of this problem. On the other hand, the number of patients with VAP is relatively low. However, our study population includes almost 1000 cases and during a whole month all patients undergoing operations were systematically included.

## Conclusions

These data, representing several European institutions, suggest that VAP is still the main cause of nosocomial infection during the postoperative period following MHS. Due to the scarcity of variables for intervention, anticipative or pre-emptive antimicrobial therapy should be explored as one of the few potential interventions to avoid VAP in the population remaining under mechanical ventilation for more than 48 hours.

## Key messages

• One or more nosocomial infections were detected in 4.4% of the patients.

• VAP was the most frequent nosocomial infection (2.1%, 13.9 episodes per 1000 days of mechanical ventilation).

• The principal microorganisms responsible for VAP in this study were: *Enterobacteriaceae *(45%), *Pseudomonas aeruginosa *(20%) and methicillin-resistant *Staphylococus aureus *(10%).

• Risk factors for VAP were: ascending aorta surgery, number of blood units transfused and need for re-intervention.

• Death was significantly more frequent in patients with VAP (35% vs 2.3%).

## Abbreviations

CABG: coronary artery bypass grafting; CI: confidence interval; CPB: cardiopulmonary bypass; CPIS: Clinical Pulmonary Infection Score; ESCMID: European Society of Clinical Microbiology and Infectious Diseases; ESGNI: European Study Group of Nosocomial Infection; EWCI: European Working Party of Cardiothoracic Intensivists; FiO_2_: fraction of inspired oxygen; ICU: intensive care unit; IQR: interquartile range; MHS: major heart surgery; NYHA: New York Heart Association; OR: odds ratio; PaO_2_: partial pressure of arterial oxygen; RR: relative risk; SD: standard deviation; VAP: ventilator-associated pneumonia.

## Competing interests

The authors declare that they have no competing interests.

## Authors' contributions

EB and JH designed the study. JH wrote the manuscript drafts. PM and GPC were responsible for the analysis of the data. All authors participated in the acquisition of the data and contributed in the writing and critical appraisal of the manuscript. All authors read and approved the final manuscript. EB critically revised the article and gave final approval to the version to be published.

## Authors' information

The following investigators collaborated in the collection of data in the different hospitals participating in the study collaborating thus with the ESGNI and the EWCI: Peter Mares (AKH University Hospital Vienna, Austria); Kirsten Eliasen (Rigshospitalet, Denmark); Durand Michel (Hopital A Michallon, CHU de Grenoble, France); Rafaele Bonato (Azienda Ospedaliera-Universita di Padova, Italy); José Antonio Moreno (Hospital Universitari Germans Trias i Pujol, Spain); Manuel Colmenero (Virgen de las Nieves, Spain); Alvarez Berceruelo (Hospital Clínico de San Carlos, Spain); Armando Maruenda (Hospital Clínico Universitario de Valencia); Primitivo Arribas (Hospital 12 de Octubre. Spain); Roberto Voces (Hospital de Cruces, Spain); José Manuel Borrallo (Meixoeiro, Spain); R. Carlos Marcos (Clínica Puerta de Hierro, Spain); Antonio Reyes (Hospital de la Princesa, Spain); Feliciano Fernández (Virgen de la Macarena, Spain); Mariano Villaseñor (Clínica Ruber, Spain); Leonardo Lorente (Hospital Universitario de Canarias, Spain); Mercedes Cuesta (Ruber Internacional, Spain); Juan Porta (Hospital La Fe, Spain); Rubén Jara-Rubio (Virgen de la Arrixaca, Spain) Johan Sellgren, Sven-Erik Ricksten (Sahlgrens University Hospital, Sweden); Daniel Schmidlin (University Hospital Zurich, Switzerland); Patrick Francioli (Centre Hospitalier Universitaire Vaudois, Switzerland).
